# Understanding the Impacts of Online Mental Health Peer Support Forums: Realist Synthesis

**DOI:** 10.2196/55750

**Published:** 2024-05-09

**Authors:** Paul Marshall, Millissa Booth, Matthew Coole, Lauren Fothergill, Zoe Glossop, Jade Haines, Andrew Harding, Rose Johnston, Steven Jones, Christopher Lodge, Karen Machin, Rachel Meacock, Kristi Nielson, Jo-Anne Puddephatt, Tamara Rakic, Paul Rayson, Heather Robinson, Jo Rycroft-Malone, Nick Shryane, Zoe Swithenbank, Sara Wise, Fiona Lobban

**Affiliations:** 1 Spectrum Centre for Mental Health Research Division of Health Research Lancaster University Lancaster United Kingdom; 2 School of Computing and Communications Lancaster University Lancaster United Kingdom; 3 Division of Health Research Lancaster University Lancaster United Kingdom; 4 IT Corporate Services Berkshire Healthcare NHS Foundation Trust Berkshire United Kingdom; 5 Survivor Research Network London United Kingdom; 6 Division of Population Health, Health Services Research & Primary Care University of Manchester Manchester United Kingdom; 7 Department of Psychology Edge Hill University Ormskirk United Kingdom; 8 Faculty of Health and Medicine Lancaster University Lancaster United Kingdom; 9 Social Statistics University of Manchester Manchester United Kingdom

**Keywords:** digital mental health, peer-to-peer support, social networking, moderation, systematic review

## Abstract

**Background:**

Online forums are widely used for mental health peer support. However, evidence of their safety and effectiveness is mixed. Further research focused on articulating the contexts in which positive and negative impacts emerge from forum use is required to inform innovations in implementation.

**Objective:**

This study aimed to develop a realist program theory to explain the impacts of online mental health peer support forums on users.

**Methods:**

We conducted a realist synthesis of literature published between 2019 and 2023 and 18 stakeholder interviews with forum staff.

**Results:**

Synthesis of 102 evidence sources and 18 interviews produced an overarching program theory comprising 22 context-mechanism-outcome configurations. Findings indicate that users’ perceptions of psychological safety and the personal relevance of forum content are foundational to ongoing engagement. Safe and active forums that provide convenient access to information and advice can lead to improvements in mental health self-efficacy. Within the context of welcoming and nonjudgmental communities, users may benefit from the opportunity to explore personal difficulties with peers, experience reduced isolation and normalization of mental health experiences, and engage in mutual encouragement. The program theory highlights the vital role of moderators in creating facilitative online spaces, stimulating community engagement, and limiting access to distressing content. A key challenge for organizations that host mental health forums lies in balancing forum openness and anonymity with the need to enforce rules, such as restrictions on what users can discuss, to promote community safety.

**Conclusions:**

This is the first realist synthesis of online mental health peer support forums. The novel program theory highlights how successful implementation depends on establishing protocols for enhancing safety and strategies for maintaining user engagement to promote forum sustainability.

**Trial Registration:**

PROSPERO CRD42022352528; https://www.crd.york.ac.uk/prospero/display_record.php?RecordID=352528

## Introduction

### Background

The World Health Organization recently identified poor supply of services as a primary barrier to mental health care worldwide [1]. Evidence-based digital approaches may alleviate demands on existing services and help meet the rising need for accessible models of psychosocial support [2]. Online mental health peer support forums may represent one such approach. Forums allow users to engage in asynchronous, text-based communication with those who share similar experiences. Common functions include the ability to start discussions, or threads, and post messages within these threads in response to others’ comments [3]. This creates opportunities to exchange information, advice, and emotional support, often within moderated online environments. Forums may focus on certain topics, such as specific mental health diagnoses, or on mental health and well-being more broadly [4].

Given that forum websites typically remain available 24 hours per day, they hold the potential to deliver accessible mental health support at scale. However, evidence of their effectiveness is mixed. While some trials of digital interventions, including forums, have shown positive effects, such as improved mood, mindfulness, and compassion [5,6], others have shown no significant impact on user well-being [7,8]. This is consistent with a review of online peer support for young people with mental health difficulties in which just 2 of 6 identified studies reporting positive effects on anxiety or smoking behaviors [4]. Factors impacting these findings include challenges with retention and engagement in online interventions [8,9], which may not reflect the way users engage with forums outside the context of intervention studies. Many such interventions include forums along with other components, such as psychoeducational materials, confounding attempts to identify specific impacts of forum use. Therefore, at present, the reasons why some online forums are more conducive to positive user experiences than others remain unclear.

Processes underpinning observed impacts of online mental health forums are likely to be multifaceted. There is qualitative evidence suggesting that some forum users derive benefit from the social connection offered by online forums [10-12]. Indeed, a conceptual model of online peer support for severe mental illness emphasized the value of online interactions for stigma reduction and increasing participants’ willingness to engage with in-person support [13]. The option to participate anonymously, a feature of many online forums, may reduce fear of judgment and promote personal disclosure related to mental health difficulties [14,15]. However, some users report that reading about mental health on the web can be distressing, and concerns have been raised about the potential of forums to proliferate harmful content [16,17]. Furthermore, it is currently unclear whether the impacts of online forums are influenced by differences in forum implementation and moderation across online contexts. Some forums are established and moderated by people with personal experience of mental health difficulties [18]. Others are delivered by mental health care providers and staffed by formally trained moderators who, depending on the service delivery model, may be health professionals or volunteers [19,20].

### Objectives

There is evidence that the use of online mental health forums is growing. For example, the open Reddit discussion board “r/depression” expanded from 314,000 users at the end of 2017 to approximately 1 million in late 2023 [21]. Increased support seeking in mental health forums in response to the COVID-19 pandemic [22] also emphasizes the need for further research on the contemporary use, safety, and effectiveness of these services. Previous reviews have identified and described mental health forum user experiences. For example, it is clear that online forum–based interventions for mental health are feasible and viewed as acceptable by most users and meet some users’ needs for informational and social support [23-25]. However, there have been recent calls for mechanistic research to better understand the processes that underpin the effects of online forums to inform innovations in forum design and implementation [26]. This study aimed to address this gap by applying realist synthesis to generate an explanatory model, or program theory, explaining the impacts of online mental health forums on users.

## Methods

This study was preregistered on PROSPERO (registration CRD42022352528) and is reported with reference to the Realist and Meta-Narrative Evidence Syntheses: Evolving Standards guidelines [27]. The synthesis progressed through 5 stages informed by realist methodological guidance [28].

### Stage 1: Define the Scope of the Synthesis

We sought evidence of the use of online mental health forums as per the definitions in Textbox 1. Instead of searching for evidence published from 1993 onward as per our research protocol, we restricted our evidence searches to between 2019 and 2023. This increased the likelihood that the included studies would reflect a similar context to the current program delivery environment, including online harms, technological functionality, and contemporary health service environments, which may serve to promote the transferability of our findings to current and future stakeholders. Our decision to search from 2019 onward was informed by a recent comprehensive review of digital health interventions that identified both a shift in the online context of health service delivery as a result of the COVID-19 pandemic and a notable increase in relevant literature in the same year [29]. It is also likely that pre-2019 articles are captured in related systematic reviews [23,24]. The scope of this synthesis was also informed by engagement with a public and patient involvement group comprising forum staff and users. The group participated in a stakeholder prioritization workshop that involved ranking and discussing the importance of research areas related to online forums. This influenced the subsequent analysis, as described in stage 5.

**Definitions of key terms.**

**Key terms and definitions used in this synthesis**

- *Mental health peer support forum*—forums were defined as on the web, primarily asynchronous text-based discussion platforms. All forum types were eligible, including those hosted on widely used social media platforms such as Reddit and those delivered as part of specifically designed interventions. To be included in this synthesis, articles must have studied a mental health peer support forum. Mental health forum was broadly defined to include any forum primarily intended to support people experiencing psychological distress, including those with specific mental health difficulties, experiencing isolation or substance misuse or addiction, or caregiving for someone with a mental health difficulty. To be eligible for inclusion, evidence sources must have described forums focused on facilitating peer-to-peer discussion.
- *User*—forum users are people who access online mental health forums to seek support for their own psychological well-being or in the capacity of an informal supporter of someone experiencing mental health difficulties. Activities in which users typically engage in online forums include reading posts, starting their own discussion topics, and responding to other users.
- *Moderator*—moderators are staff or volunteers with oversight responsibilities within online forums. While these roles vary across contexts, they typically include facilitating discussions within forums, moderating content, enforcing rules, and providing support directly to users by responding to their posts. Any moderation type was eligible, including moderation by volunteers, health professionals, or community members with personal experience of the mental health difficulties discussed on the forum. Forums with no peer-to-peer discussion, such as those in which service users interact solely with mental health professionals, were not eligible for inclusion.
- *Program*—the intervention or service under investigation, which in realist research is typically a health or social care program [28]. In this synthesis, “program” refers to online mental health forums.
- *Context*—a factor that determines the activation or triggering, or the strength of activation or triggering, of a given mechanism. Contexts are varied and can include factors at psychological, social, economic, and institutional levels [28].
- *Mechanism*—the hidden force that causes a program to work, defined as the ways in which a participant responds to a program [28]. We used the distinction between mechanism resource (what is offered by a program) and reasoning (how users respond to what is offered) to further elaborate our analysis [30].
- *Outcome*—the expected and unexpected results of a program [28].
- *Context-mechanism-outcome configuration* (CMO)—a CMO is a heuristic used in realist research to articulate causal insights regarding how a particular program generates outcomes, with reference to the operation of mechanisms in specific contexts [27].
- *Program theory*—an explanation of how the program under investigation works. In this synthesis, CMOs represent individual program theories. We also outline an “overarching program theory,” which refers to the integration of individual program theories to create an explanatory model of how the entire program under investigation operates.

### Stage 2: Develop Initial Program Theories

Initial program theories are preliminary accounts of how a program is expected to work, which are subsequently refined through the synthesis process [28]. To support initial program theory development, we held a multistakeholder workshop comprising forum users, staff, and researchers with expertise in digital mental health. Attendees completed a group activity focused on designing a hypothetical forum and explored its potential impacts, mechanisms, and challenges. The research team used these discussions to generate a list of initial ideas of how forums work, including a series of “if, then” statements used to support causal reasoning in the early stages of realist research [31]. An overview of the ideas and initial program theories generated is presented in Multimedia Appendix 1.

### Stage 3: Search for Evidence

Searches were conducted on the following health and social science databases from January 2019 to May 2023: PsycINFO, MEDLINE, CINAHL, Academic Search Ultimate, Embase, Scopus, and Web of Science. The search strategy was developed in collaboration with an information specialist at Lancaster University and was informed by a sensitivity analysis. This involved checking whether searches returned key articles previously identified by the study team as relevant to the research question. Database searches were supplemented with gray literature searches on Google, the TRIP medical database, Overton, the International Clinical Trials Registry, the National Grey Literature Collection, ProQuest, and the National Health Service Knowledge and Library Hub. The full search strategy is available in Multimedia Appendix 2 [5,13,16,32-38].

### Stage 4: Selection and Appraisal

Documents were assessed for eligibility against the following criteria: (1) documents referred to a peer online mental health forum as per the definition in Textbox 1; (2) documents referring to users who were adults or young people, defined as >50% of participants being aged ≥13 years; (3) full texts available in English; and (4) any document type or study design.

Documents were ineligible for inclusion if they met any of the following criteria: (1) documents focused on a online platform that did not primarily support asynchronous text-based group discussion. Examples include interventions whose primary functions were direct instant messaging, live chat, or image sharing; (2) documents focused on an intervention principally aimed to support the self-management of a physical health difficulty and did not have an explicit focus on psychological distress; and (3) documents focused on an intervention aimed at supporting the practice of mental health professionals.

Titles and abstracts were screened in the web-based systematic review platform Rayyan (Rayyan Systems Inc) [39]. Screening was completed by a team of 9 researchers who initially independently screened 100 articles and then met to identify discrepancies and refine the screening procedure. Each team member screened a separate batch of articles. The team met weekly and made decisions on articles collaboratively where ambiguity existed regarding their eligibility. Articles that passed title and abstract screening were reviewed in full by a second reviewer to confirm their eligibility against the inclusion criteria, during which articles were appraised with reference to realist-informed principles of rigor and richness [40], where evidence sources are judged with respect to their relevance to theory development. We applied an inclusive assessment of rigor based on the “good enough” test [41]. Therefore, articles were included if they were sufficiently transparent to allow the reader to understand how the data had been generated and if they were credible given the methodology used. That is, the methods used in each evidence source were congruent with the results and conclusions drawn from them. To judge richness, we adapted the “traffic-light” system [42] for judging the usefulness of evidence sources for their potential contribution to program theory development, with screeners making judgments of low, moderate, or high usefulness. Highly useful evidence sources were eligible for inclusion in the synthesis. Judgments regarding rigor and usefulness were double-checked by a second reviewer before data extraction. The full-text screening instructions are presented in Multimedia Appendix 3 [40,43].

### Stage 5: Data Extraction and Synthesis

Data were extracted describing key study characteristics, including year of publication, title, authors, and a description of the forum and moderation. We then extracted data relevant to program theory development. To do this, researchers copied data segments from eligible documents into an Excel (Microsoft Corp) spreadsheet and added an analytical code. Analytical codes articulated how the data segment contributed to program theory development, with a specific focus on identifying contexts, mechanisms, and outcomes as per the definitions in Textbox 1. Data analysis ran in parallel. This initially involved developing candidate context-mechanism-outcome configurations (CMOs) informed by concepts identified in the initial stakeholder workshops. CMOs were then iteratively refined by integrating insights from data segments and analytical codes obtained from the data extraction process. To promote analytical rigor, a core team of analysts including the lead interviewer (PM) and researchers with expertise in online mental health forum research (FL and HR) and realist methods (AH) met regularly to review extracted data and collaboratively develop each individual CMO in group discussion. The CMOs were reviewed by the wider research team, comprising academics, clinicians, forum moderators, and lived experience researchers with broad experience in digital mental health and peer support.

Data from the existing literature identified in the database searches predominantly focus on the experience of forum users, with many articles using qualitative methods, including interviews. Therefore, we supplemented this literature with exploratory interviews with other key stakeholders likely to provide insights into the positive and negative impacts of online mental health forums (the topic guide and participant demographics are available in Multimedia Appendix 4). Participants were recruited from UK institutions involved in the delivery of online mental health forums as part of a funded project to investigate mental health forum use in the United Kingdom [44]. Participants were purposively sampled via email advertisements circulated to forums partnered with the project. They included “forum hosts” (n=5), who were clinicians with oversight roles within organizations that host mental health forums and clinical academics who have designed and delivered online forum–based interventions, and forum moderators (n=13). The sample size was informed by both the research team’s judgment of data sufficiency in answering the research question and a pragmatic consideration regarding the resources available for this study The interviews focused on articulating participants’ views on the impacts of online forum use (outcomes), the processes underlying these impacts (mechanisms), and the factors influencing the presence of those processes or the extent to which they occurred (contexts). The interviews were conducted in parallel to data extraction from eligible studies and used the same analytic procedure. That is, transcribed data were copied into the data extraction document with a code identifying how the data informed CMO development.

Individual CMOs were then grouped thematically into 5 theory areas reflecting the research priorities set in the stakeholder prioritization workshop. These were how learning in forums benefits user mental health (theory area 1—mental health self-efficacy), factors impacting safety (theory area 2—psychological safety), factors impacting the use of forums and other services (theory area 3—service use), the role of forum moderators (theory area 4—moderation), and how connecting with others online impacts user well-being (theory area 5—social connection). Following CMO refinement, figures were generated indicating links between program theories to provide overarching explanations for how forums generate intended and unintended impacts. Finally, 3 members of a public and patient involvement group were invited to individually review and provide feedback on an interim version of the analysis before the final write-up. This group is facilitated by the research team and comprises forum users, staff, and people with lived experience of mental health difficulties. Therefore, group members had a previous relationship with the research team and knowledge of the aims of this study.

### Ethical Considerations

This study received ethical approval from Solihull Research Ethics Committee on 20 June 2022 (IRAS314029).

## Results

### Included Evidence Sources

**Figure 1:**
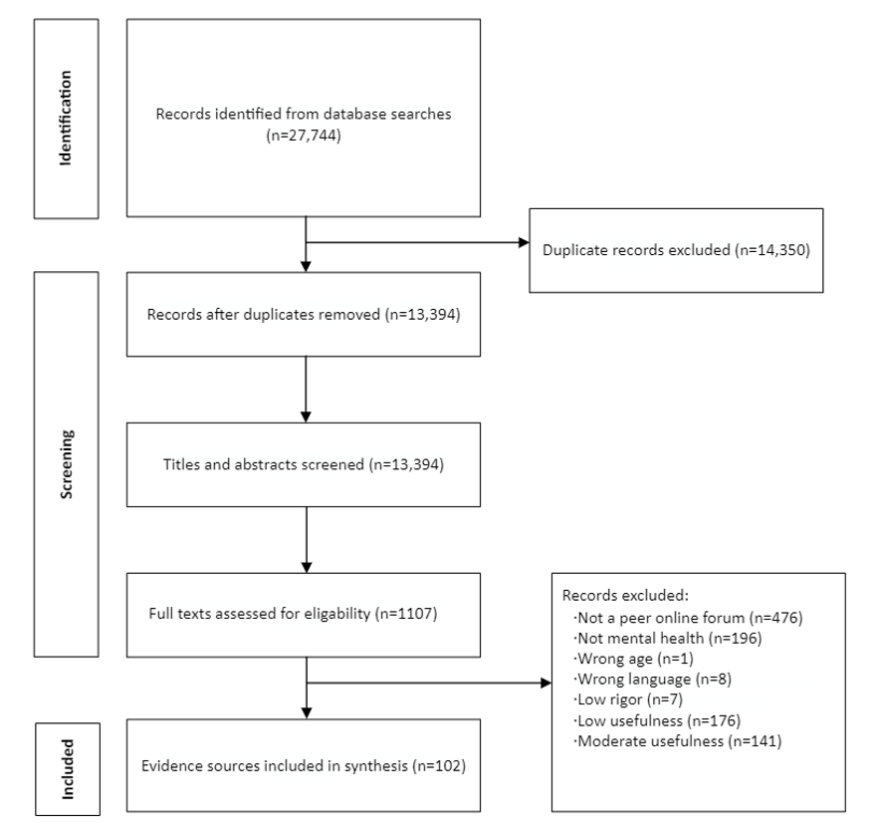
Process of evidence identification.

The process of evidence identification (Figure 1) led to the inclusion of 102 documents. The characteristics of the included evidence sources are presented in Multimedia Appendix 5 [18,25,32,33,44-81].

### Program Theories

**Figure 2:**
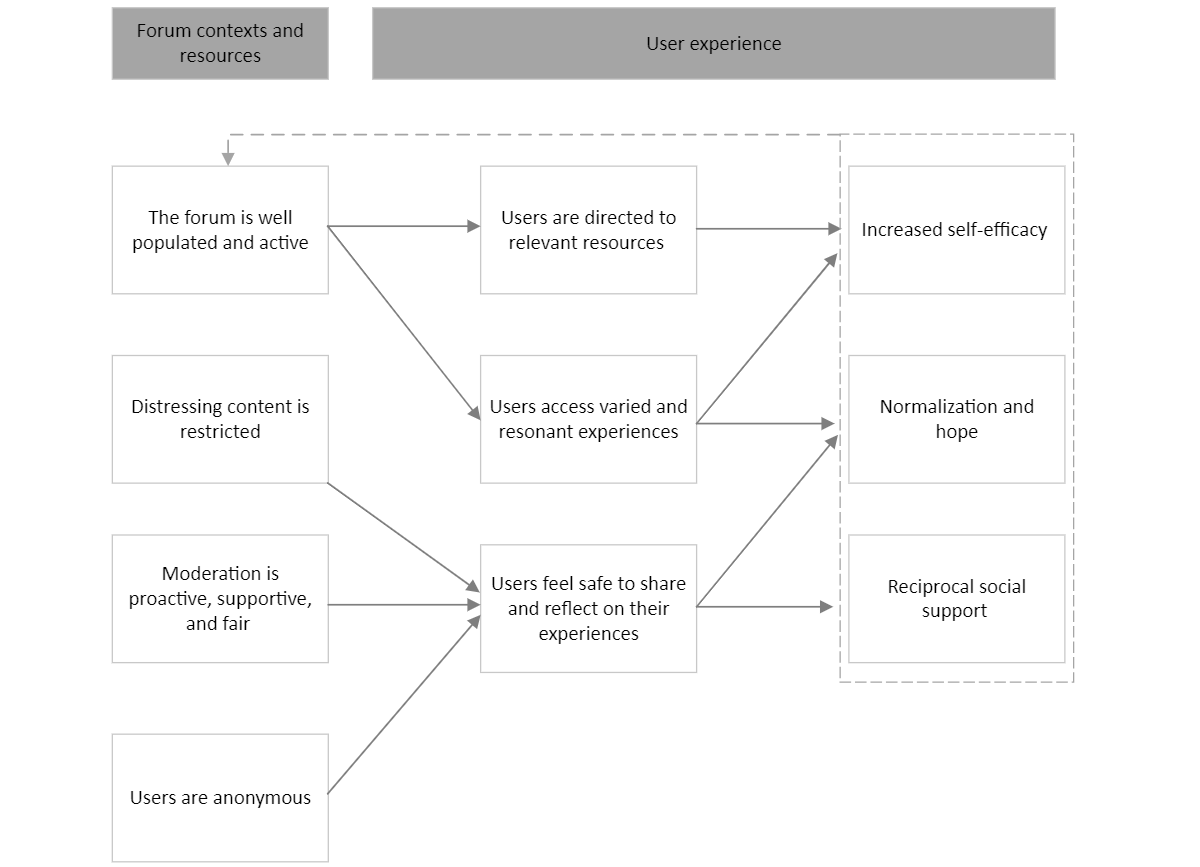
Overarching program theory—intended impacts of online mental health peer support forums.

**Figure 3:**
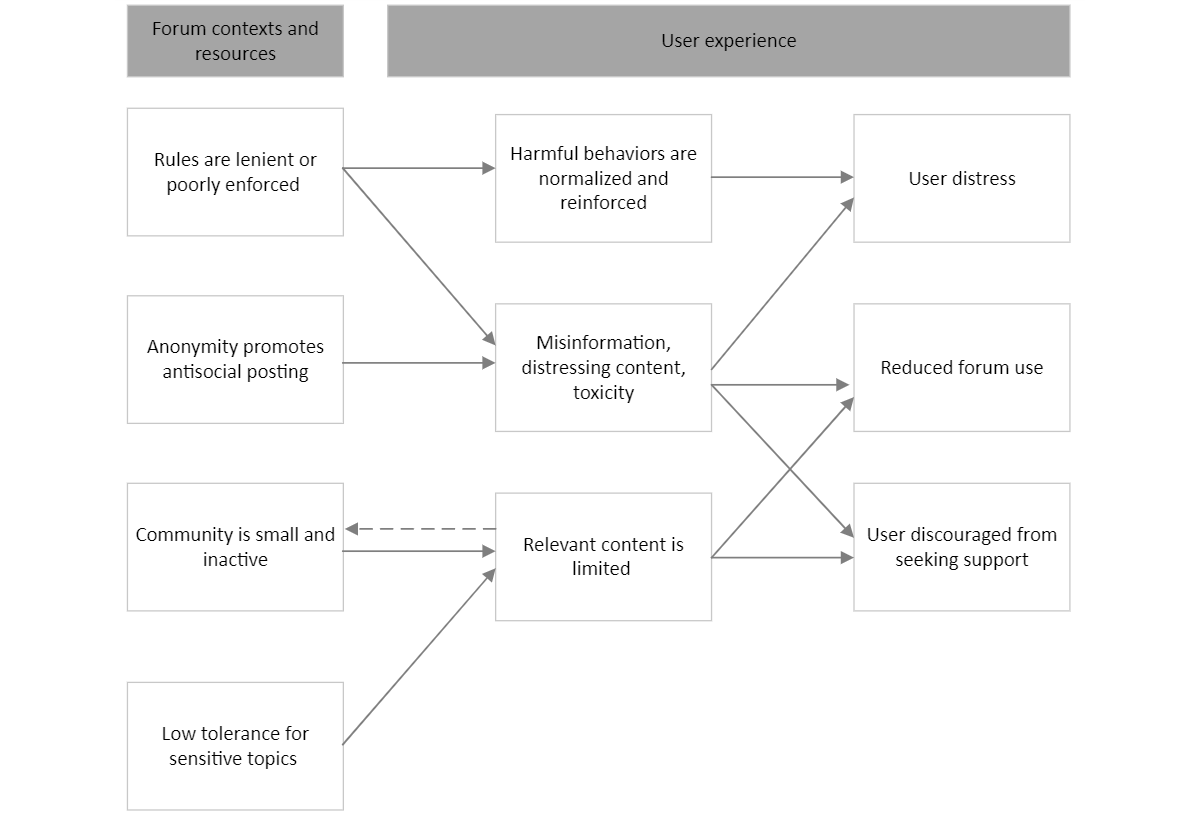
Overarching program theory—unintended impacts of online mental health peer support forums.

Data analysis produced 22 CMOs (Textbox 2) in 5 theory areas. Figures 2 and 3 present overarching program theories representing intended and unintended impacts, respectively. A narrative summary of the program theories is presented in this section with reference to illustrative evidence. Quotes with citations are from evidence sources identified in the literature search. Quotes from interviews are indicated by the participant’s role and number, for example, forum moderator 1.

**Context-mechanism-outcome configurations (CMOs) by theory area.**

**Theory area 1: mental health self-efficacy**

- CMO 1: in well-populated and active forums that are clearly organized (context) to allow users to find posts and receive responses that are personally relevant (mechanism—resource), users will be more likely to identify credible and actionable information that they can use to better manage their mental health (mechanism—reasoning), promoting mental health self-efficacy (outcome).
- CMO 2: when users feel safe to share their mental health experiences (context) with others whom they perceive to be nonjudgmental and as having relevant experiences (mechanism—resource), they will use the forum to reflect on their circumstances and integrate others’ perspectives into their own (mechanism—reasoning), resulting in novel and more hopeful ways of making sense of their mental health experiences (outcome).

**Theory area 2: psychological safety**

- CMO 3: posts detailing personal experiences of potentially harmful behaviors (eg, self-injury and restrictive eating; context) that frame them as helpful (mechanism—resource) may normalize and reinforce their use (mechanism—reasoning), increasing the likelihood of users adopting these behaviors (outcome).
- CMO 4: when seeking support for issues that others may find distressing (context), users are more likely to post in forums that have ways to flag the potentially distressing nature of their experiences (eg, tags, trigger warnings, or a separate subforum; mechanism—resource). This provides reassurance that posts will not inadvertently cause harm to others (mechanism—reasoning), increasing the likelihood that users will use the forum to seek support (outcome). Other users are less likely to be exposed to distressing content (mechanism—reasoning), reducing potential distress in the wider community (outcome).
- CMO 5: for users making an original post (context), the absence of a response or responses that are unrelated to the original post (mechanism—resource) will prompt feelings of being ignored or misunderstood (mechanism—reasoning). This leads to increased isolation (outcome) and reduces forum engagement (outcome).
- CMO 6: those yet to post to forums may be concerned about feeling exposed or receiving negative responses if they share their experiences (context). Observers who see others receiving constructive and respectful responses (mechanism—resource) will be reassured of the safety of posting to the forum (mechanism—reasoning), increasing the likelihood that they will actively participate in discussions (outcome).
- CMO 7.1: negative social consequences of discussing mental health difficulties, including shame and stigma (context), are overcome by forum anonymity (mechanism—resource), which disinhibits (mechanism—reasoning) users discussing their experiences, leading to greater self-disclosure (outcome).
- CMO 7.2: because users’ personal identities are hidden (context), they are insulated from the negative social consequences of rule breaking (mechanism—resource). This can have a disinhibiting effect on some users (mechanism—reasoning), making them more likely to engage in antisocial behavior such as bullying (outcome), reducing safety for other users (outcome).
- CMO 8: open online forums with no ways to flag distressing content, poor moderation, or lenient rules (context) are more likely to expose users to posts detailing users’ highly distressing circumstances, misinformation, and “toxic” discussions (mechanism—resource), which can contribute to distress (mechanism—reasoning) and disengagement from the forum (outcome).

**Theory area 3: service use**

- CMO 9: when users experience barriers to in-person mental health care, such as stigma, poor service availability, or living in a rural area (context), accessible online forums (mechanism—resource) are seen as convenient sources of support (mechanism—reasoning), increasing forum use in those experiencing these barriers (outcome).
- CMO 10: in cases in which forums are populated with people who have positive experiences of mental health services (context) and who share these experiences with a view to encouraging other users to seek help (mechanism—resource), readers will feel more confident in approaching those services (mechanism—reasoning), increasing use of other forms of mental health support (outcome).
- CMO 11: design features (mechanism—resource) that inhibit the autonomous and competent use of forum technology (mechanism—reasoning) decrease users’ motivation for engagement (outcome), particularly in cases in which that technology is novel for individual users (context).
- CMO 12: when users who are yet to seek alternative (non–forum-related) mental health support (context) are exposed to negative comments about those sources of support, such as mental health services (mechanism—resource), they will be more skeptical of the potential value of those services (mechanism—reasoning) and, therefore, less likely to approach them (outcome).
- CMO 13: in cases in which a supportive online community (context) provides the emotional and informational support a user requires (mechanism—resource), that user will feel that their needs are met sufficiently by that community, reducing the perceived need for alternative support (mechanism—reasoning) and, therefore, the use of other mental health services (outcome).

**Theory area 4: forum moderation**

- CMO 14: when initially accessing an online forum (context), friendly support with how to use the site and the presence of moderators who are seen to promote positive engagement (mechanism—resource) generates confidence in using the forum (mechanism—reasoning), increasing subsequent engagement (outcome).
- CMO 15: in cases in which forums are moderated and users post to the forum (context), moderator responses that are timely, show empathy and understanding, are personalized to the content of users’ original posts, and invite further discussion (mechanism—resource) will lead users to feel heard and supported (mechanism—reasoning), prompting further engagement with the forum (outcome).
- CMO 16: when forum moderators intervene in forum discussions to restrict or delete users’ rule-breaking posts (context), doing so in a way that demonstrates consistency and makes site rules clear (mechanism—resource) will mean that users view their actions as fair and unobtrusive (mechanism—reasoning), promoting trust and safety among the wider user base (outcome).
- CMO 17: when forums have low tolerance for discussions of potentially distressing issues (context), moderators are more likely to delete comments referencing related topics such as self-injury (mechanism—resource). While this may promote a sense of safety (mechanism—reasoning) and engagement (outcome) for some users, those whose posts are deleted may feel that this action infringes on their autonomy and ability to seek support (mechanism—reasoning), prompting attempts to avoid moderation (eg, by tangential references to banned material; outcome) or seek support in less restrictive forums (outcome).

**Theory area 5: social connection**

- CMO 18: when forums bring together people with similar personal experiences (context), users have access to posts that resonate with their circumstances (mechanism—resource). This normalizes their mental health experiences and validates their own reactions to similar situations (mechanism—reasoning). This can reduce self-stigma (outcome) and provide a sense of belonging (outcome).
- CMO 19: when forums provide users with a reliable source of support (context), the ability to interact with the community when needed (mechanism—resource) decreases users’ reliance on in-person informal support (mechanism—reasoning), reducing perceived burdensomeness on friends and relatives (outcome).
- CMO 20: users who share their personal experiences on the web (context) derive satisfaction (outcome) from the knowledge that their posts help others (mechanism—reasoning), particularly when others express gratitude (mechanism—resource).
- CMO 21: when forum users post messages (context) and receive timely, constructive, and empathetic responses from other users (mechanism—resource), they will feel recognized and understood (mechanism—reasoning). This will contribute to a sense of connection (outcome) with the online community (outcome), increasing forum engagement (outcome).
- CMO 22: when users disclose lived experience (context), other users are more likely to view the user as authentic (mechanism—reasoning). This makes users more likely to share their own experiences in response, generating reciprocal and mutually supportive conversations and relationships (outcome).

### Theory Area 1: Mental Health Self-Efficacy

Promoting mental health self-efficacy, a belief in one’s own capacity to manage mental health, is a common goal of forum-based interventions [45,82]. This is enabled by providing users with access to credible and actionable information of personal relevance (CMO 1). Examples include lived experience expertise regarding how to manage certain situations, such as caring for a relative in distress, and techniques users can apply to support their own recovery [46,47]:

...when they share information about coping strategies, I “cherry-pick” the things that suit me most.

Users may identify this information in the repository of existing threads or post their own requests for information on specific issues. Finding relevant information and receiving helpful responses relies on an active community that generates new content:

...we did also run the project in small Trusts [regional health services] and we tried to have forums in the Trust, and it didn’t work at all and it’s basically because you need a big enough population that there’s always activity otherwise people won’t go to it.

Forum host 1

As one forum host noted, grouping users into forums with a narrow focus can limit the breadth of information generated:

...you want a 24/7 digital, vibrant community where any concern that someone brings will be mirrored by somebody else or at some stage in life...to avoid running people into dead ends, very, very narrow, let’s say, identifications, we try to keep the groupings as large as possible because that’s where you get that diversity of experience and perspective.

Forum host 2

CMO 2 highlights the value of collaborative sense making within forum conversations. In the context of a safe and nonjudgmental online space, peer discussion facilitates an exploration of personal difficulties through the lens of others’ experiences [32,48,83]. Such discussions allow for a broadening of users’ understandings of mental health, which can serve to reframe their own [49] by, for example, reconceptualizing a shared difficulty as something that can be managed [32]:

I remember there was one guy that used to say, it does get better. It gets a lot better, and I know that this is an awful feeling now, but it does get better and that was really big. That was life-changing.

Therefore, forums offer opportunities to reflect on and experiment with new personal narratives, a process that can lead to greater clarity and self-awareness [47]:

...sharing experiences you get different viewpoint and more insight, this makes you think more seriously, like, “ah, that could be the same for me, or maybe that’s a pitfall for me too.”

Exposure to others’ perspectives, particularly those describing progress in managing mental health, could lead users to adopt more positive expectations [46]:

...posters glimpsed the possibility of recovery for the first time; this prompted the initial act of hoping as they came to believe that this transformation was possible for themselves.

### Theory Area 2: Psychological Safety

Several articles identified a sense of safety as foundational to positive forum experiences [32,50,83]. Aspects of psychological safety may include the confidence to use the platform without being exposed to distressing content or negative judgment. Evidence sources also identified factors that could undermine this sense of safety. For example, some communities allow users to share details of potentially harmful and distressing experiences, including self-injury and restrictive eating (CMO 3) [51-53]. Descriptions of these behaviors may frame them as helpful and understandable ways to manage emotional distress, creating a culture that normalizes and encourages their use. This has been described as a “functional paradox” [53], where those in suicidal distress seek a form of social support that increases the risk of adverse events or where those stigmatized by issues such as eating disorders look for understanding peer communities that perpetuate the issues for which users require support [52].

While some users derive benefit from simply reading online forums [54], others may desire interaction but not feel sufficiently comfortable to engage in conversation directly. For example, some users are reluctant to post about their own distressing circumstances for fear of causing harm to others in the community (CMO 4) [55]:

I guess I didn’t even post things, like thoughts that I might be having...life’s not worth living, I feel so hopeless, I feel like I don’t have a future. I wouldn’t say stuff like that, because I thought it would be too triggering for other people.

This may be mitigated by the inclusion of subsections or content notices to allow potential readers to avoid harmful content and promote community safety [84]. Moreover, receiving no reply can cause frustration and undermine the sense of connection that draws people to forums (CMO 5) [55,56]. Seeing others receive timely and encouraging responses can promote confidence in the forum and help those considering posting become active forum participants (CMO 6) [57].

There is broad recognition that anonymity facilitates dialogue within online forums (CMO 7.1) [47,53,58-60]. Online anonymity alleviates concerns linked to in-person help seeking [82]:

The desire for anonymity was often a result of fear and stigma and could be more prominent in certain communities and cultures....“You are anonymous. And you can leave when you want, whereas if you go to a group you tend to be there for at least a polite amount of time.”

Therefore, forums provide an outlet for users to express their “true self” [61] and broach topics that may be difficult or seemingly impossible to address in person:

One lady disclosed domestic abuse. She hadn’t disclosed it to her midwife, GP, health visitor who’d she’d all seen in the flesh etcetera, but she felt safe enough to disclose it to us because it was anonymous, she was anonymous.

Forum moderator 1

However, this same feature can lead to different user responses. Therefore, CMO 7.2 represents a rival theory [31], stating that anonymity may render interactions impersonal and limit consequences for “toxic” or “trolling” behaviors [61], including hostile comments and harassment [18,62]:

I also volunteer on a different online community with anonymity and I find there’s more conflict of people thinking you can literally say whatever and not so supportive.

Forum moderator 1

Harmful content and antisocial behaviors may be more likely to occur in communities that have little or no moderation (CMO 8). Clear and visible rules for what cannot be discussed on the forum, proactive monitoring for rule-breaking content, and the restriction of users’ access to forums where rules are broken are important for promoting a supportive culture and mitigating risks to the wider user base [53]. Such risks may include being exposed to stigmatizing messages, misinformation, or highly distressing content [63]. Cultivating a community characterized by supportive communication that also provides the opportunity for users to openly express their difficulties has been described as a “balancing act” for moderators [64]. Rules need to be sufficiently stringent to limit harmful posting but not to the extent that they are a deterrent to engagement:

I think guidelines are important. I think they need to be easy to understand and not make it feel like, not make someone feel scared for breaking a rule sort of thing.

Forum host 1

A related concern for promoting forum safety relates to the potential cumulative effect of being exposed to a large volume of particularly distressing posts, which can be detrimental for users [47]:

Well, you can feel overwhelmed by it all. I had mixed feelings. On the one hand, I felt relief because I could share my experiences. But on the other hand...all the new posts—I thought, “This is not good. I’m too preoccupied with the forum and worry too much about others right now.”

### Theory Area 3: Service Use

Motivations for using online peer support forums provide insights into what works for whom (CMO 9). Motivations include a lack of understanding from friends or family members and associated challenges with in-person help seeking [32,61,65], such as fear of judgment from social contacts or health services. Reflecting on an eating disorder support forum, one participant noted the following [52]:

...we need a place where we can feel accepted, appreciated, and safe. And this is it.

Indeed, limited access to in-person sources of support can lead some users to engage with online communities [32,66]. Alternatively, users who are yet to seek in-person support can be empowered to do so through interactions with peers (CMO 10). Therefore, forums may represent a “stepping stone” [47] to further support, which can result from direct encouragement, the sharing of specific advice regarding how to access support, and a growing confidence with discussing mental health with others [67-69]. Reflecting on their own forum use, a moderator noted the following:

I would never have got into services had it not been from peer support online with people encouraging me to tell my parents what was going on, tell my teachers, go to the GP, helping me to even write the letter that I wrote to my GP expressing what was going on.

Forum moderator 2

Forum design that supports competent self-directed use, such as easy-to-use interfaces, is an important determinant of ongoing service engagement [70] (CMO 11). Features users may find beneficial include easy navigation, app integration, and the inclusion of capabilities such as emojis [58]. Conversely, frustrating technological issues can undermine perceived convenience:

...having a community that’s easy to use is important so I think some feedback we’ve had...when you first join our community, we’ve got so many subforums that it looks quite busy and overwhelming so something we want to do is condense it a little bit.

Forum host 3

There are also contexts in which forum use may lead users to be less likely to seek in-person support. The stakeholder workshop highlighted the possibility that forum use may alleviate pressures on health services by meeting users’ needs without them requiring other support (CMO 12). However, in some circumstances, users may be exposed to posts describing unsuccessful attempts to access services or negative experiences in health care that could discourage readers from seeking support (CMO 13) [62,71], an issue noted by one interviewee:

...it could be quite triggering if someone’s having an experience that’s very similar to yours and it could be quite depressing I suppose just thinking, “god, I’m struggling but these people are struggling even more...” No one’s got an answer to this. Quite hopeless. I think it could lead to a bit of hopelessness about how bad the system is...

Forum host 1

### Theory Area 4: Forum Moderation

Moderators play important roles in users’ early forum experiences, where their guidance helps users engage with the community and sets expectations for what the forum can provide (CMO 14) [25,70]. Being consistently visible can offer reassurance that interactions will remain positive and supportive, as one moderator highlighted:

I think also what’s important is moderator presence, so they know that we’re around and looking after the community and kind of replying to reports quite quickly I think yeah it just helps people feel safe, able to reach out.

Forum moderator 3

The style and content of moderators’ online posts are likely to influence users’ satisfaction and ongoing engagement with the forum (CMO 15). As identified by one forum host, effective moderators achieve a balance between sharing relevant information and offering understanding:

...it was a very skilled approach, so it was thoughtful, it was deep—extensive. It was informed and it was sort of tapping into both the empathic side of it and the need for information, so it wasn’t just providing information and it wasn’t, “Oh I’m sorry you feel like that,” I think there was a really good balance between empathy and information.

Forum host 2

Users also valued timely responses, which both helped address current difficulties [48,82] and mitigated against forum users disengaging from the community:

I think there is data as well that says like 50% of people don’t come back if they haven’t had a reply within 24 hours.

Forum host 3

When removing content that contravenes forum rules, moderators must balance a desire to facilitate open peer discussion with a need to ensure the appropriateness of the content shared on the platform [32]. Making decisions transparent and consistent helps establish boundaries for what users can expect to do and see within a forum and may mitigate the risk that users feel unfairly treated (CMO 16) [55]. However, moderators may face challenges when implementing rules related to potentially harmful behaviors, such as self-injury or restrictive eating (CMO 17). Users affected by these issues may feel that their need for support is undermined by strict content policies, particularly in cases in which online forums represent one of the few safe spaces to seek help. Regarding an eating disorder forum banned by a host website, a former user recalled the following:

...someone had posted what to do if you feel like you’re going to binge, what to do if you feel like you can’t eat today. I would go and read that actively, like, “Oh, here’s some reminders for myself,” and now it’s gone, and I can’t access that.

Restricting community discussion in this way can lead to frustration [55]; isolation [72,84]; and attempts to navigate these boundaries, for example, limiting how open a user is about their difficulties [32] or using novel terminology to overcome restrictions on what can be discussed [51]. While such moderator decisions are often made in the interest of the wider community, individual support may be deprioritized:

I’ve worked for a few other online communities...they kind of banned people right away if they were in crisis which I found really difficult to do because it didn’t feel very fair that they were reaching out for support and they get banned instantly.

Forum host 3

This illustrates how organizations and moderators negotiate an often sensitive and challenging responsibility to balance the interests and safety of individuals against impacts on the wider community.

### Theory Area 5: Social Connection

The social connection offered by online mental health forums facilitates several distinct positive impacts. Others’ accounts of similar circumstances validate users’ own thoughts and behaviors [73], contributing to a recognition that they are not isolated in their mental health experiences (CMO 18) [62]:

...knowing you aren’t alone, that you’re not crazy or lazy, that other people go through the same thing every day, is a strangely comforting thing to experience.

This normalizing experience can reduce self-stigma and blame [46]:

I’ve always felt that my responses to what had happened seemed abnormal and crazy, so I feel reassured reading that it’s okay to react the way I did. I can now work on finding healthier solutions to my issues.

Other elements of user responses likely to promote perceived social support include them being offered in a timely way when users most need help [47,68], constructive suggestions for problem-solving [45,74], and empathetic communication [32,75] (CMO 19). For example, in one forum, this support was represented by statements such as “I’m glad you’re here,” “So much love to you,” or “This whole thing is a nightmare.” Emotional support was also provided relatively prominently in the form of encouragement, which would frequently occur in short interjections such as “you can do it, mama” or “There IS light at the end of the tunnel!!” [76]. Community members may derive benefit from offering such support (CMO 20) [50,55,77], particularly in cases in which users are motivated to support others through situations that they have personally experienced [62]:

I find great joy in helping others find resources and helping them to learn about this condition and its comorbid conditions, as well as relating my personal experiences to theirs so that they, in turn, don’t feel so alone.

Within forum threads, the presence of personal narratives promotes the authenticity of users’ requests for support and prompts reciprocal and mutually beneficial sharing in response (CMO 21) [78,79]. In this way, relationships develop within the community, improving users’ perceptions of being socially supported [47]:

...I was trying to focus on solving my own problems until I saw that users were helping each other. I realized I could also benefit from their support. I began typing up my personal story. I got positive replies and then also started to respond to others.

This process not only occurs through conversations about mental health, but also, as with in-person relationships, connections develop through exchanging updates on daily life, venting frustrations, and discussing personal interests [50,83]. Providing an example of ongoing community support, one moderator recalled the following:

...they [users] come back to us and they say, “yeah, the problem I had with my friend, it’s all sorted now. It’s great,” and all the young people, all their peers are like, “Ah that’s so amazing. I remember your post and it sounded so awful. I’m so proud of you for sorting that out,” so they get really positive validation from their peers.

Forum moderator 4

In cases in which online mental health forums provide users with a regular source of social support, their perception of being a burden on in-person social contacts may diminish (CMO 22) [32,47,80].

## Discussion

### Principal Findings

This paper presents a novel program theory highlighting the potential for safe and active online peer support forums to promote mental health self-efficacy through access to actionable information and the opportunity to explore personal difficulties with nonjudgmental peers (theory area 1). It points to the importance of psychological safety (theory area 2) in facilitating positive experiences and identifies barriers to safety, including exposure to distressing content and concerns about posting. Motivations for forum use include stigma and difficulties accessing in-person services, whereas the nature of forum experiences may shape users’ perceptions of those wider services (theory area 3). Proactive and supportive forum moderation is important for creating a space for dialogue (theory area 4) where users can engage in mutual and reciprocal social support, which can lead to reduced isolation and a sense of connection (theory area 5).

### Comparison With Prior Work

The findings reported in this paper are consistent with but also extend those of the conceptual model of online peer support by Naslund et al [13] for people with severe mental illness. Their model highlights stigma, isolation, and fear of judgment as precursors to forum use. Positive forum experiences are proposed to result in reduced stigma; increased help seeking; and participant activation, defined as learning from and acting upon others’ experiential knowledge. By drawing on a diverse evidence base, this synthesis highlights how similar processes underlie positive forum user experiences in a range of contemporary mental health–related contexts, including for family carers [56] and among people with physical health conditions seeking psychological support [62]. This implies that, as with in-person peer support, key processes underlying effective online peer support, including experiential knowledge sharing and reciprocally supportive relationships [81], may represent transdiagnostic mechanisms that are present across service delivery modalities. However, the results of this synthesis emphasize that positive impacts are context dependent. Outcomes including mental health self-efficacy and social connection occur within the context of supportive and vibrant forum cultures proactively managed to minimize rule-breaking content and behaviors. Therefore, when successfully implemented, forums become places for what has been termed “infomotional support” [25], which reflects the combination of simultaneous empathetic emotional support and practical information highly valued by forum users.

This synthesis highlights the importance of the ways in which potentially distressing topics such as suicidal thoughts and behaviors are permitted and managed within peer online forums. Research into online forums specifically focused on suicide prevention provides insights into challenges associated with implementing these services. Consistent with the findings reported in this paper, clear expectation setting was a key component of a social networking intervention for youth suicidal ideation [80]. On agreeing to terms of use, users were informed of forum rules, including restrictions on suicide-related discussion, and the intermittent nature of moderation. The platform used automatic keyword detection to block posts about suicide and prioritized safety over complete anonymity by collecting personal and clinical details used to raise risk concerns with health services. Qualitative research with the users of this forum suggests that, while this proactive approach to risk management did contribute to a safe and supportive environment, there are tensions inherent in restricting discussions of suicidal experiences [55]. Participants noted the value of this policy, particularly for limiting access to distressing content, yet others experienced frustration at not having a space to express their experiences linked to suicidal behavior and noted that such restrictions could perpetuate stigma. Moreover, a suicide prevention forum in the Netherlands implemented proactive moderation to remove descriptions of self-injury and provide ongoing signposting to crisis support services [53]. However, survey data indicated that, while 35% of the participants felt better after use, 12% felt worse and 13% used the forum to find information about suicide methods, with the authors calling into question the service’s benefit-to-harm ratio. This evidence highlights both the importance of initial forum design that accounts for potential harms and also the necessity of ongoing evaluation to understand how the content generated on forums impacts user experience and well-being over time.

Our findings emphasize the importance of forum activity for creating the conditions in which users find relevant information and social support. As indicated in the program theory presented in Figure 2, positive forum experiences can serve to create a feedback loop, sustaining online communities via a “network effect” of accelerating online connections [85]. Conversely, inactive forums provide little incentive for users to return, and if limited use is prolonged, there is likely to be a critical point at which forums cease to operate as intended. Therefore, online forum hosts may wish to consider both how to attract new users and how to promote engagement in current forum participants. Regarding the former, forum designers could be guided by previous intervention research, which indicates that a combination of online methods and offline strategies is required to optimize participant recruitment, including social media promotion and endorsement by third-sector and health service providers [33,86]. Regarding sustaining activity, this synthesis suggests that moderators are key to within-forum engagement. This mirrors previous review findings highlighting the facilitative role of forum moderators, who may take on activity-promoting tasks including inducting users into the platform and ensuring that the forum features up-to-date content [25]. A related direction for further research that could extend these findings relates to differences in moderator roles and users’ perceptions of moderation across different forum contexts. For example, it is currently uncertain to what extent the status of the moderator as a volunteer, health professional, or peer with lived experience influences factors such as forum activity, user disclosure, and forum culture.

The findings of this synthesis raise several important implications for future research and practice. The evidence included in this synthesis highlights the breadth of settings in which forums have been used to support different mental health problems. Despite differences in delivery contexts, the findings of this study indicate key factors underpinning forum safety and effectiveness, including rule enforcement, proactive and interpersonally sensitive moderation, and the importance of sustaining user engagement to facilitate peer interaction. This suggests that a core set of design features and implementation steps may improve the use and helpfulness of online forums aimed at supporting mental health. As previously stated [44], the findings of this synthesis will inform “best practice” design guidance that aims to advance standards for forum development and evaluation. Relatedly, the findings reported in this paper highlight a range of potential psychosocial processes that could inform future empirical work. For example, future research may seek to investigate the extent to which impacts of forum use on mental health are explained by improvements in perceived social support and mental health self-efficacy. The specific program theories reported in this synthesis are being assessed in a mixed methods realist evaluation with forum users from several UK mental health communities [44].

### Limitations

This study has some limitations. The stakeholders interviewed for this synthesis were recruited from UK-based mental health organizations. Participant experiences may not reflect those in other settings and locations, such as user-led communities hosted worldwide. Furthermore, the evidence sources and the subsequent analysis focused primarily on positive and negative experiences of those using forums, with little attention paid to why people who are offered access to forums decline to use them. Better understanding the reasons for nonuse is an important goal of further research with the potential to address barriers to engagement.

### Conclusions

Online mental health forums are becoming increasingly prominent resources for people seeking support. This synthesis of recent evidence and stakeholder interviews provides a program theory to explain how positive impacts, such as an improved ability to manage mental health and fulfilling social connection, are more likely to occur in the context of well-organized, regulated, and active forums that provide a supported space for open discussion. Forum design and implementation should consider the limits of what specific forums can and should be used for and how potentially distressing content that falls beyond these limits can be managed in ways that mitigate risks to individuals and broader forum communities.

## Appendix

Multimedia Appendix 1 Initial program theories.

Multimedia Appendix 2 Search strategy.

Multimedia Appendix 3 Full-text screening instructions.

Multimedia Appendix 4 Interview participant demographics and topic guide.

Multimedia Appendix 5 Characteristics of the included studies.

## Abbreviations

CMO: context-mechanism-outcome configuration

## Data Availability

The data sets generated during and analyzed during this study are not publicly available. This is to protect participant confidentiality as the qualitative interview data used in this study cannot be fully anonymized. Data are available from the corresponding author on reasonable request.
