# Supplementary material for: Understanding the Impacts of Online Mental Health Peer Support Forums: Realist Synthesis
Source: JMIR Ment Health. 2024 May 9;11:e55750. doi: 10.2196/55750 (PMC11117133; doi:10.2196/55750)
Supplement: Multimedia Appendix 1 [file mental_v11i1e55750_app1.docx]

**iPOF initial programme theories**

Elements of CMO configurations identified in stakeholder workshop activities.

**Contexts**

*Individual*​

- Levels and times of use​
- Alignment of member lived expertise with content of forum​.
- Member trauma levels linked to issues being discussed​.
- Purpose of joining the forum- specific advice vs general support​
- Demographic alignment with forum ​
- Mood / receptiveness / anger when reading the posts​.
- Previous experiences in forums​
- Early posts (or well into journey)​
- General levels of use and familiarity- consider lurking as well as posting​.
- Motivation for using the forum​.
- Levels of social anxiety in offline world ​
- Digital literacy​

*Organisational*​

- Size and heterogeneity of forum​
- Culture of forum – how far distress is allowed to be expressed. ​
- Accessibility and ease of use of forum- e.g. ability to search 24/7, emojis, app etc. ​
- Moderator personality style​
- How risks are identified and managed ​
- Anonymity levels – privacy / public​
- Perceived host and endorsers (are it seen as credible?)​
- Open / self-referral / prof referrer required​
- Levels of moderation (pre read, % of posts responded to, frequency of hiding removing posts etc.)​
- Functioning and reliability of the forum site​
- Use of incentives – like, followers, comments etc​
- Is forum embedded within other intervention components e.g. toolboxes, blogs etc. etc.​
- Sharing of data and consent processes

**Mechanisms – resources**

- Volume of replies​
- Speed of replies​
- Distribution of posts across members (or dominated by superusers)​
- Emotional valence of replies​
- Quality of content in replies​
- Timeliness of moderation ​
- Skill of moderation​
- Levels of distress / risk expressed in posts. ​
- Perceived credibility of host / endorser​
- signposting​

**Mechanism - responses**

- How safe they feel to share openly – including trust in moderation process​.
- learning new knowledge and skills​
- Shaping of behaviour via feedback from other members- testing things out in safe environment​
- Perceived social support​.
- Normalization of experiences​
- Feeling heard and validated – vs overfocus on distress and no boundaries. ​
- Extent to which identify with other members of the forum and “like them”- seeking further contact​.
- Perceived authenticity / humanness of other forum members​
- Ability to see change over time in others – generating hope​.
- Recognize early signs of relapse and opportunity for early intervention based on feedback from others and / or watching the process in others. ​​

Outcomes

- Levels of use / engagement​
- Types of use / engagement (patterns, content, how open)​
- Wellbeing / distress​
- Isolation / loneliness / connectedness​
- Daily functioning​
- Mental health literacy​
- (perceived) stigma – self and other​.
- Help seeking behaviors including use of other services – could increase or decrease​.
- Identity in relation to mental health​
- Hope and optimism / hopelessness at lack of change​
- Meaning in past experiences​
- Empowerment - Self efficacy in relation to managing mental health​.
- Perceived social support​.
- Openness and trust in other ​
- Greater self-awareness / understanding​

​

**Examples of initial ‘If, then’ statements generated based on stakeholder workshops.**

- If a member discloses a personal / sensitive experience in the forum and they receive no responses, within a specific time period, or the responses invalidate or contradict the experience, then they are likely to feel more misunderstood, less connected and are less likely to post in future.
- If a new member reads a post early on that resonates with their own experience, then they are more likely to feel connected to the group and to visit again. The more similar the person posting is recognized to be (age, gender, ethnicity, MH diagnosis), the more connected they feel.
- If members read posts about how things have improved for other people with a shared challenge, then they are likely to feel more hopeful about their own recovery, but only if the posts also include specific details about how this improvement has happened and the other people posting are considered to be similar (MH diagnosis?) to them. This is because they can then see a route or map to recovery that they could follow.
- Conversely, if members read posts about things getting worse for another member, then they will feel less hopeful for their own recovery, but only if there is no detail about the reasons for the worsening, so they cannot understand why this has happened. If reasons are stated that do not affect the member OR the other poster is seen to be quite different in demographic characteristics, then this will not impact them as they won’t fear the same thing happening to them.
- Some people will go from being ‘lurkers’ to advice seekers to advice givers. Presumably, if someone gets helpful responses and feels comfortable and part of the community, they may in some cases feel involved and confident enough to provide advice for others.
- Members who lack confidence in the online world, may feel more able to engage with online groups because they are anonymous, unseen, unheard etc. This is likely to be true for people who perceive they have some kind of abnormality that will lead them to be rejected in real world settings. As they connect, and build confidence, they feel more EMPOWERED to explore relationships in the outside world and the connection is generalized.
- Members who regularly share personal experiences in response to a request for information or support from other members of the forum, may feel that  they have come a long way from where they were when they were experiencing similar challenges, and that they are now in a better place in which their life has more meaning e.g. through work, relationships etc. This may only happen if the experiences they share are sufficient in the past.
