# Supplementary material for: Understanding the Impacts of Online Mental Health Peer Support Forums: Realist Synthesis
Source: JMIR Ment Health. 2024 May 9;11:e55750. doi: 10.2196/55750 (PMC11117133; doi:10.2196/55750)
Supplement: Multimedia Appendix 2 [file mental_v11i1e55750_app2.docx]

**iPOF Search strategy**

**Database searches**

We developed a free text search string for each of our concepts (‘online’, ‘peer/forum’, and ‘mental health’) for use on relevant disciplinary databases hosted on the EBSCO platform (PsychINFO, Medline, Academic Search Ultimate and CINAHL). Free-text search strings were applied to title and abstract fields using ‘OR’. Concepts were combined using ‘AND’. We also searched for relevant database-specific subject terms.

We developed a second free text search string for use on large, non-EBSCO multidisciplinary databases (EMBASE, SCOPUS, and Web of Science). We combined this search string with databases specific subject terms in EMBASE only, as SCOPUS and Web of Science do not support this function. We therefore applied free text search strings to the ‘keywords’ field in SCOPUS and Web of science.

Searches were limited to English language articles. The search period was from 1993-present, with 1993 representing a key date in the development of the internet (the release of HTML) and the technological capacity to host online forums.

**PsychINFO (search fields: title OR abstract OR subject terms)**

**Free text string**

Online

"online" OR ( ("web" OR "computer" OR "virtual" OR "online" OR "internet" OR "digital") N3 ("commun*" OR "interven*" OR "based" OR "platform*" OR "social network*")) OR "bulletin board system*" OR "e-communit*" OR "ecommunit*" OR "e-health" OR "ehealth" OR "eintervention*" OR "e-intervention*" OR "mhealth" OR "m-health" OR ("mobile" N3 ("application*" OR"app" OR apps"))

Peer/forum

"communit*" OR "e-communit*" OR "ecommunit*" OR "forum*" OR "peer" OR "peer-to-peer" OR ("support*" N3 ("group*" OR "network*" OR "psychological" OR "social" OR "online")) OR (("moderat*") N3 ("forum*" OR "group*" OR communit*"))

Mental health

(("affective" OR "attention deficit" OR "adjustment" OR "body image" OR "mental" OR "dissociative" OR "addict*" OR "eating" OR "gambling" OR "hoarding" OR "personality" OR "panic" OR "obsession" OR "post-traumatic" OR "posttraumatic" OR "mood" OR "eating" OR "emotion*" OR "psycho*")

N3 ("disord*" OR "problem*")) OR "anxiety" OR "*phobia" OR "anorexia" OR "bipolar" OR "mental illness*" OR "mental health" OR "depress*" OR "mental distress*" OR (("drug*" OR "substance*" OR "alcohol") N3 ("misuse" OR "use" OR "abuse")) OR "e-mental health" OR "gender dysphoria " OR "loneliness" OR "self-injur*" OR "pro-ana" OR "proana" OR "psychiatric" OR "psychosis" OR

"schizo*" OR "self-harm*" OR "selfharm*" OR ("sleep" N3 ("disrupt*" OR "disorder*")) OR "suicid*"

**Subject terms**

#### Online

DE “Online Social Networks” OR DE “Online Community” OR DE “Social Media” OR DE "Online Behavior" OR DE "Internet" OR DE "Computer Mediated Communication" OR DE "Electronic Communication" OR DE "Digital Technology"

#### Peer Support / Forum

DE “Peers” OR DE “Peer Relations” OR DE “Peer Counseling” OR DE "Interpersonal Relationships" OR DE "Friendship" OR DE "Role Models" OR DE "Peer Tutoring" OR DE "Interpersonal Interaction" OR DE "Social Support" OR DE "Emotional Support" OR DE "Social Connectedness" OR DE "Social Interaction" OR DE "Social Networks" OR DE "Support Groups" OR DE "Social Communication"

#### Mental Health

DE “Mental Health” OR DE “Digital Mental Health Resources” OR DE “Mental Disorders” OR DE “Mental Status” OR DE “Abnormal Psychology” OR DE “Clinical Psychology” OR DE “Psychopathology” OR DE “Psychiatric Symptoms” OR DE “Serious Mental Illness”

**Medline (title OR abstract OR subject terms)**

**Free text string**

Online

"online" OR ( ("web" OR "computer" OR "virtual" OR "online" OR "internet" OR "digital") N3 ("commun*" OR "interven*" OR "based" OR "platform*" OR "social network*")) OR "bulletin board system*" OR "e-communit*" OR "ecommunit*" OR "e-health" OR "ehealth" OR "eintervention*" OR "e-intervention*" OR "mhealth" OR "m-health" OR ("mobile" N3 ("application*" OR"app" OR "apps"))

Peer/forum

"communit*" OR "e-communit*" OR "ecommunit*" OR "forum*" OR "peer" OR "peer-to-peer" OR ("support*" N3 ("group*" OR "network*" OR "psychological" OR "social" OR "online")) OR (("moderat*") N3 ("forum*" OR "group*" OR communit*"))

Mental health

(("affective" OR "attention deficit" OR "adjustment" OR "body image" OR "mental" OR "dissociative" OR "addict*" OR "eating" OR "gambling" OR "hoarding" OR "personality" OR "panic" OR "obsession" OR "post-traumatic" OR "posttraumatic" OR "mood" OR "eating" OR "emotion*" OR "psycho*")

N3 ("disord*" OR "problem*")) OR "anxiety" OR "*phobia" OR "anorexia" OR "bipolar" OR "mental illness*" OR "mental health" OR "depress*" OR "mental distress*" OR (("drug*" OR "substance*" OR "alcohol") N3 ("misuse" OR "use" OR "abuse")) OR "e-mental health" OR "gender dysphoria " OR "loneliness" OR "self-injur*" OR "pro-ana" OR "proana" OR "psychiatric" OR "psychosis" OR

"schizo*" OR "self-harm*" OR "selfharm*" OR ("sleep" N3 ("disrupt*" OR "disorder*")) OR "suicid*"

**Subject terms**

#### Online

(MH “Online Systems”) OR (MH “Online Social Networking”) OR (MH “Internet-Based Intervention”) OR (MH “Internet”) OR (MH “Social Media”) OR (MH “Digital Technology”)

#### Peer Support / Forum

(MH “Peer Group”) OR (MH “Friends”) OR (MH “Interpersonal Relations”) OR (MH “Social Support”) OR (MH “Social Interaction”) OR (MH “Social Networking”) OR (MH “Psychosocial Support Systems”)

#### Mental Health

(MH “Mental Health”) OR (MH “Mental Health Recovery”) OR (MH “Mental Disorders”) OR (MH “Mentally Ill Persons”) OR (MH “Psychopathology”) OR (MH “Psychology, Clinical”)

**CINAHL (title OR abstract OR subject terms)**

**Free text string**

Online

"online" OR ( ("web" OR "computer" OR "virtual" OR "online" OR "internet" OR "digital") N3 ("commun*" OR "interven*" OR "based" OR "platform*" OR "social network*")) OR "bulletin board system*" OR "e-communit*" OR "ecommunit*" OR "e-health" OR "ehealth" OR "eintervention*" OR "e-intervention*" OR "mhealth" OR "m-health" OR ("mobile" N3 ("application*" OR"app" OR "apps"))

Peer/forum

"communit*" OR "e-communit*" OR "ecommunit*" OR "forum*" OR "peer" OR "peer-to-peer" OR ("support*" N3 ("group*" OR "network*" OR "psychological" OR "social" OR "online")) OR (("moderat*") N3 ("forum*" OR "group*" OR communit*"))

Mental health

(("affective" OR "attention deficit" OR "adjustment" OR "body image" OR "mental" OR "dissociative" OR "addict*" OR "eating" OR "gambling" OR "hoarding" OR "personality" OR "panic" OR "obsession" OR "post-traumatic" OR "posttraumatic" OR "mood" OR "eating" OR "emotion*" OR "psycho*")

N3 ("disord*" OR "problem*")) OR "anxiety" OR "*phobia" OR "anorexia" OR "bipolar" OR "mental illness*" OR "mental health" OR "depress*" OR "mental distress*" OR (("drug*" OR "substance*" OR "alcohol") N3 ("misuse" OR "use" OR "abuse")) OR "e-mental health" OR "gender dysphoria " OR "loneliness" OR "self-injur*" OR "pro-ana" OR "proana" OR "psychiatric" OR "psychosis" OR

"schizo*" OR "self-harm*" OR "selfharm*" OR ("sleep" N3 ("disrupt*" OR "disorder*")) OR "suicid*"

**Subject terms**

#### Online

(MH “Online Services”) OR (MH “Online Systems”) OR (MH “Online Social Networking”) OR (MH “Internet-Based Intervention”) OR (MH “Internet”) OR (MH “World Wide Web”) OR (MH “Social Media”) OR (MH “Electronic Bulletin Boards”) OR (MH “Digital Technology”)

#### Peer Support / Forum

(MH “Peer Counseling”) OR (MH “Peer Group”) OR (MH “Peer Assistance Programs”) OR (MH “Support, Social”) OR (MH “Support, Psychosocial”) OR (MH “Social Networks”) OR (MH “Interpersonal Relations”) OR (MH “Friendship”) OR (MH “Support Groups”)

#### Mental Health

(MH “Mental Health”) OR (MH “Mental Disorders”) OR (MH “Mental Disorders, Chronic”) OR (MH “Psychopathology”) OR (MH “Clinical Psychology”)

**Academic Search Ultimate (title OR abstract OR subject terms)**

**Free text string**

Online

"online" OR ( ("web" OR "computer" OR "virtual" OR "online" OR "internet" OR "digital") N3 ("commun*" OR "interven*" OR "based" OR "platform*" OR "social network*")) OR "bulletin board system*" OR "e-communit*" OR "ecommunit*" OR "e-health" OR "ehealth" OR "eintervention*" OR "e-intervention*" OR "mhealth" OR "m-health" OR ("mobile" N3 ("application*" OR"app" OR "apps"))

Peer/forum

"communit*" OR "e-communit*" OR "ecommunit*" OR "forum*" OR "peer" OR "peer-to-peer" OR ("support*" N3 ("group*" OR "network*" OR "psychological" OR "social" OR "online")) OR (("moderat*") N3 ("forum*" OR "group*" OR communit*"))

Mental health

(("affective" OR "attention deficit" OR "adjustment" OR "body image" OR "mental" OR "dissociative" OR "addict*" OR "eating" OR "gambling" OR "hoarding" OR "personality" OR "panic" OR "obsession" OR "post-traumatic" OR "posttraumatic" OR "mood" OR "eating" OR "emotion*" OR "psycho*")

N3 ("disord*" OR "problem*")) OR "anxiety" OR "*phobia" OR "anorexia" OR "bipolar" OR "mental illness*" OR "mental health" OR "depress*" OR "mental distress*" OR (("drug*" OR "substance*" OR "alcohol") N3 ("misuse" OR "use" OR "abuse")) OR "e-mental health" OR "gender dysphoria " OR "loneliness" OR "self-injur*" OR "pro-ana" OR "proana" OR "psychiatric" OR "psychosis" OR

"schizo*" OR "self-harm*" OR "selfharm*" OR ("sleep" N3 ("disrupt*" OR "disorder*")) OR "suicid*"

**Subject terms**

#### Online

DE “ONLINE chat” OR DE “INTERNET forums” OR DE “ONLINE comments” OR DE “ONLINE social networks software” OR DE “ONLINE social networks” DE “SOCIAL networking mobile apps” OR DE “SOCIAL media” OR DE “WEBSITES” OR DE “COMPUTER bulletin boards” OR DE “INTERNET” OR DE “ONLINE comments”

#### Peer Support / Forum

DE “FORUMS” OR DE “INTERNET forums” OR DE “ONLINE comments” OR DE “PEER counseling” OR DE “ONLINE chat” OR DE “PEERS” OR DE “INTERNET forums software” OR DE “FORUMS (Discussion & debate) – Social aspects” OR DE “PEER relations” OR DE “VIRTUAL communities” OR DE “INTERPERSONAL communication” OR DE “PEER communication” OR DE “PEER relations” OR DE “FRIENDSHIP” OR DE “SOCIAL networks” OR DE “SOCIAL exchange” OR DE “INTERNET friendship” OR DE “SOCIAL support” OR DE “SUPPORT groups” OR DE “INTERNET content moderation” OR DE “VOLUNTEER workers in mental health”

#### Mental Health

DE “MENTAL health” OR DE “MENTAL illness” OR DE “PATHOLOGICAL psychology” OR DE “PEOPLE with mental illness” OR DE “CLINICAL psychology” OR DE “ABNORMAL psychology” OR DE “VOLUNTEER workers in mental health”

**EMBASE (title OR abstract OR subject terms)**

**Free text**

Online

online OR internet OR web-base* OR "web base*" OR webbase* OR e-communication OR "e communication" OR ecommunication

Peer/forum

peer OR peers OR forum* OR communit* OR ecommunit* OR e-communit* OR "e communit*" OR ((digital OR online) NEAR/3 (group* OR network* OR support*))

Mental health

((mental OR psych*) NEAR/3 (health OR disorder* OR illness* OR distress* OR well-being OR wellbeing OR "well being")) OR psychotherap* OR self-injur* OR "self injur*" OR selfinjur* OR "self harm*" OR self-harm* OR selfharm*

**Subject terms**

Online

online social network/ OR internet/ OR online system/ OR social media/ or digital technology/ or web-based intervention

Peer/forum

peer group/ OR peer counseling/ OR interpersonal communication/ OR friendship/ OR friend/ OR social interaction/ OR social connectedness/ OR social network/ OR social support/ OR social connectedness/ OR social interaction/ OR support group/ OR psychosocial care/

Mental health

mental health/ OR mental disease/ OR abnormal behavior/ OR clinical psychology/ OR psychiatric diagnosis/ OR mental patient/

**Scopus (title OR abstract OR keywords)**

**Free text**

Online

online OR internet OR web-base* OR "web base*" OR webbase* OR e-communication OR "e communication" OR ecommunication

Peer/forum

peer OR peers OR forum* OR communit* OR ecommunit* OR e-communit* OR "e communit*" OR ((digital OR online) NEAR/3 (group* OR network* OR support*))

Mental health

((mental OR psych*) NEAR/3 (health OR disorder* OR illness* OR distress* OR well-being OR wellbeing OR "well being")) OR psychotherap* OR self-injur* OR "self injur*" OR selfinjur* OR "self harm*" OR self-harm* OR selfharm*

**Web of Science (title OR abstract OR keywords)**

Online

online OR internet OR web-base* OR "web base*" OR webbase* OR e-communication OR "e communication" OR ecommunication

Peer/forum

peer OR peers OR forum* OR communit* OR ecommunit* OR e-communit* OR "e communit*" OR ((digital OR online) NEAR/3 (group* OR network* OR support*))

Mental health

((mental OR psych*) NEAR/3 (health OR disorder* OR illness* OR distress* OR well-being OR wellbeing OR "well being")) OR psychotherap* OR self-injur* OR "self injur*" OR selfinjur* OR "self harm*" OR self-harm* OR selfharm*

Sensitivity search

Gold standard papers were used to inform a sensitivity test of the above search strategy. Search strings/subject terms were modified until all available articles were found in each database in which they are indexed (searches were then restricted to 2019-present):

- Qualitative Exploration of the Potential for Adverse Events When Using an Online Peer Support Network for Mental Health: Cross-Sectional Survey. Easton et al 2017
- The Effectiveness of an Online Support Group for Members of the Community with Depression: A Randomised Controlled Trial. Griffiths et al. 2012
- An Online, Moderated Peer-to-Peer Support Bulletin Board for Depression: User-Perceived Advantages and Disadvantages. Griffiths et al 2015
- Start, stop, and continue: Preliminary insight into the appeal of self-injury e-communities. Lewis et al. 2016
- A direct to public peer support programme (Big White Wall) versus web-based information to aid self-management of depression and anxiety: results and challenges of an automated randomised controlled trial. Morriss et al 2021
- Examining thematic similarity, difference, and membership in three online mental health communities from reddit: A text mining and visualization approach. Park et al 2018
- Moments of Change: Analyzing Peer-Based Cognitive Support in Online Mental Health Forums. Pruksachatkun et al 2019
- The future of mental health care: peer-to-peer support and social media. Naslund et al 2016
- Social Connection and Online Engagement: Insights From Interviews With Users of a Mental Health Online Forum. Smith-Merry et al 2019
- Digital atmospheres: affective practices of care in Elefriends. Tucker et al 2017

**Grey literature search strategy**

Our grey literature search strategy is intended to retrieve unpublished and non-academic evidence relevant to the synthesis. We focused on UK-based evidence sources (e.g. NHS websites) in order to retain relevance to context in which the study is being run. Based on limited evidence returned by unsystematic scoping searches we extended the data limit 2012.

Free text search string:

(online forum* or online communit*) AND (“mental health” OR “psycho*” OR “disorder” OR “mental illness”)

Databases to search:

- Overton
- TRIP
- Google  (using ‘inurl’ function)
- .ac
- .nhs
- .gov
- .org
- .edu
- Allcatsrgrey
- International clinical trials registry
- ProQuest
- NHS Knowledge and Library Hub

Included Document types:

- Blogs
- Policy documents (UK only)
- Research studies (including third sector reports, conference papers etc) not present in database searches
- Training documents and moderation manuals
- Theses

Limiters:

- 2019 - present
- English language online
