# Supplementary material for: Understanding the Impacts of Online Mental Health Peer Support Forums: Realist Synthesis
Source: JMIR Ment Health. 2024 May 9;11:e55750. doi: 10.2196/55750 (PMC11117133; doi:10.2196/55750)
Supplement: Multimedia Appendix 3 [file mental_v11i1e55750_app3.docx]

**iPOF full-text screening**

**Stage 1: full-text checking against inclusion criteria**

If unsure – respond ‘maybe’ and bring the query to the next group meeting.

**1. Is the article written in English**

Yes: continue screening

No: add the Rayyan label “FTS not English” and stop screening

(note that FTS = full-text screening)

**2. Was the article published 2019 onwards?**

Yes: continue screening

No: add label “FTS wrong date” and stop screening

**3. Is the publication type eligible for inclusion?**

Yes: continue screening

No: add label “FTS wrong publication type” and stop screening

Explanation:

Include: all research, books, policy documents, commentaries, training manuals, blogs

Exclude: Non-text documents (podcasts, videos), corrections or retractions, book reviews, documents that only refer to talks that have not been transcribed t

**4. Is the paper focussed on young people or adults?**

Yes: continue screening

No: add label “FTS wrong age” and stop screening

Explanation:

The evidence primarily (>50%) relates to those aged 13+. If age is not explicitly mentioned, try to determine participant age range from the context of the paper

**5. Does the article refer to the use on an online peer forum?**

Yes: continue screening

No: add label “FTS not POF”

Explanation:

POF definition: online platform for text-based discussion by users with shared experience. The platform must be asynchronous and access to peer comments should not be time restricted.

Include:

- Text-based social media which is set up specifically to support mental health
- Group chats

Exclude:

- if discussion is restricted to direct messages
- if the forum is used primarily to communicate with non-peer groups (e.g. health professionals)
- Twitter
- Youtube/Instagram - or other image-based platforms

**6. Is the article about a forum that aims to support some aspect of mental health?**

Yes: continue screening

No: add label “FTS not mental health”

Explanation:

Mental health is defined broadly to include any aspect of psychological distress. The forum must primarily aim to support some aspect of mental health. Those that primarily aim to physical health management are ineligible.

**Include**:

- Any mental health problem/diagnosis
- Loneliness
- Substance misuse
- Carers of people with mental health problems
- Groups focused on potentially harmful behaviours (e.g. self-harm)

**Exclude**:

- Neurological conditions without mental health focus
- Learning disability without mental health focus
- Mental health professional practice
- Non-problematic substance use

**Stage 2: assessment of relevance, rigour and richness**

*For further contextualising information, see relevant publications e.g. Dada et al., 2023*

**1. Is the evidence in this document rigorous (or ‘good enough’)?**

If yes, continue screening

If no, add label: FTS not rigorous

Explanation:

The question of rigour relates to whether the piece of evidence is good enough to be included in the review. When making this judgment, consider two following questions:

a. **Is the evidence credible?**

Considered whether the claims made within the source of evidence are plausible given the type publication and methodology used. Credibility is enhanced when the argument made is coherent, logical, and has clear explanatory value.

**AND**

b. **Is the evidence trustworthy?**

It should be clear how the evidence has been generated. Sufficient detail should be provided to allow the reader to understand how the authors have concluded what they have about peer online forums. Where data are presented, the methodological approach should be described in detail to allow the reader to make judgments about whether the study has been conducted appropriately.

**2. Is the evidence useful (usefulness = relevance + richness)?**

Evidence should be useful for developing theories regarding the impact of peer online forums for mental health on forum users, for whom these impacts are generated, why, and in what contexts

We are particularly interested in how peer online forums support mental health recovery via the following process:

- Connectedness

- Hope and optimism

- Identity

- Meaning

- Empowerment

We are also interested in factors that impact forum use and how the use of online forums may impact the way that forum users interact with other support services.

Useful data can come from ANY part of the document including introduction (eg rationale for the forum) or the discussion. This is different to traditional reviews, which generally focuses only on the results section.

Please rate the evidence source’s usefulness and choose an appropriate label:

Highly useful:

- FTS high usefulness – users
- FTS high usefulness – mods
- FTS high usefulness – users/mods

Moderately useful:

- FTS moderate usefulness – users
- FTS moderate usefulness – mods
- FTS moderate usefulness – users/mods

Low usefulness:

- FTS low usefulness

Advice on determining usefulness:

**High usefulness**: The evidence presented is likely to support realist programme theory development. High usefulness evidence facilitates the generation of programme theories by providing in depth descriptions and/or conceptual explanations relevant to the research question. It includes detail regarding contextual factors important to the functioning of online forums, and/or proposes mechanisms related to how they work and/or suggests impacts of forum use/moderation.

Highly useful evidence may occur throughout the evidence source or may be limited to short but insightful sections of text (as per Pawson’s (2007) conceptualisation of ‘nuggets’ of useful information). It may relate to any combination (or individual component) of a context, mechanisms, or outcome configuration:

**Contexts**: Important elements of the situation in which the online forum is delivered. Contexts determine whether and to what extent mechanisms are triggered. They could occur at different conceptual levels and may include user characteristics such as their motivation to use a forum; forum level factors including its functionality; or broader contextual factors like digital literacy.

**Mechanisms**: The way in which people respond to what is offered by the forum. This could include the way people think about what they see on the forum and/or their emotional reactions. For example, a feeling of having your mental health experiences normalised (mechanism) may result from seeing others’ similar experiences on the forum.

**Outcomes**: The psychological/behavioural changes that result from the firing of mechanisms in a specific context. This could include changes such as improvements in mood, perceived ability to manage mental health problems etc.

**Moderate usefulness:** The evidence is somewhat useful. It provides some information that may assist with generating programme theories/some elements of CMO configurations. However, the evidence is less rich, lacks explanatory depth, and may be more descriptive compared with high usefulness pieces of evidence. For this reason, the evidence is limited in the extent to which it can be used to develop, support, refute, or refine programme theories.

**Low usefulness**: The evidence provides very limited detail related to why, for whom and in which contexts peer online forums generate outcomes of interest. It may have passed screening because it is an article about online mental health forums, but lacks any detail regarding how they work. This form of evidence is unlikely to add a significant value to the review.

References

Dada, S., Dalkin, S., Gilmore, B., Hunter, R., & Mukumbang, F. C. (2023). Applying and reporting relevance, richness and rigour in realist evidence appraisals: Advancing key concepts in realist reviews. *Research synthesis methods*, *14*(3), 504-514.

Pawson, R. (2006). Digging for nuggets: how ‘bad’ research can yield ‘good’ evidence. *International Journal of Social Research Methodology*, *9*(2), 127-142.
