# Supplementary material for: Understanding the Impacts of Online Mental Health Peer Support Forums: Realist Synthesis
Source: JMIR Ment Health. 2024 May 9;11:e55750. doi: 10.2196/55750 (PMC11117133; doi:10.2196/55750)
Supplement: Multimedia Appendix 4 [file mental_v11i1e55750_app4.docx]

**IPOF interview participant demographics and topic guide**

**Participant demographics**

| **Role and participant number** | **Age** | **Gender** | **Ethnicity** |
| --- | --- | --- | --- |
| Host 1 | 46-55 | Male | White/White British |
| Moderator 1 | 16-25 | Female | White/White British |
| Moderator 2 | 16-25 | Female | White/White British |
| Moderator 3 | 26-35 | Male | White/White British |
| Moderator 4 | 36-45 | Female | Black/African/Caribbean/Black British |
| Moderator 5 | 16-25 | Female | Asian/Asian British |
| Host 2 | 46-55 | Female | Other Ethnic Group |
| Moderator 6 | 26-35 | Female | White/White British |
| Host 3 | 56-65 | Female | White/White British |
| Moderator 7 | 26-35 | Female | White/White British |
| Moderator 8 | 26-35 | Female | White/White British |
| Host 4 | 56-65 | Male | White/White British |
| Moderator 9 | 26-35 | Male | White/White British |
| Moderator 10 | 26-35 | Female | White/White British |
| Moderator 11 | 26-35 | Female | Mixed/Multiple Ethnic Groups |
| Moderator 12 | 26-35 | Female | White/White British |
| Host 5 | 46-55 | Female | White/White British |
| Moderator 13 | 26-35 | Female | White/White British |

**Topic guide**

The topic guide should be amended in line with the kind of participant being interviewed. Participants are likely to be forum hosts, moderators, and researchers.

Suggested topics and example questions (select as appropriate). These topics remain flexible as per the semi-structured interview method.

*Topic 1: Details about the participant and their role in relation to forums*

- Can you tell me a bit about your role?
- Are you [hosting/moderating/researching] a specific type of forum?
  - target users
  - design
  - role of moderators

*Topic 2 – Pattern of role*

- - How did you come to have this role?
  - How long have you been in this role?
  - How much time do you spend in this role?
  - how has pattern of use changed over time? why?
  - if left – why left?

*Topic 2: Sharing experiences*

- Can you describe what you think happens in peer online mental health forums [or specifically the one they are involved with]?
  - what kinds of things are discussed? how? why? by who?
  - What do you see as the role of a forum?
  - What do you like about forums? Anything you don’t like or wish was different?

*Topic 3: Outcomes/impacts*

- Why do you think some NHS Trusts / charities offer peer online mental health forums?
  - what impact might it have on their organisation? (explore positives and negatives)
  - why? and how? (explore mechanism of all impacts and contextual factors that make this more or less likely to occur)
- What do you think people can gain from being in a forum? Explore positive and negative impacts.
- For each impact identified
  - how do you think this happens? (explore underlying mechanism and any contextual factors that allow this to happen)
  - what things make it more likely to happen? what might prevent this happening? (explore triggering contextual factors)
  - are there any particular kinds of people more likely to experience (each impact)? Or any kinds of people who might not get this? Why?

*Topic 4: engagement*

- What do you think engages people to use forums?
  - what might trigger someone to post?
  - why do some people use them a lot and others only once or twice?
- Why do you think some people observe but don’t post?
  - what do they get out of the forum?
- Why do some people become “superusers”?
  - what do you think might be the impacts of that? how? why and in what context?
- Why do you think some people become forum moderators?
  - what do you think might be the impacts of that? How? why and in what context?

*Topic 5: Designing best policy and practice. The aim of the study is to develop some best policy and practice tools. These might include 1) how to design a forum; 2) training and support for moderators; 3) information for commissioners and health professionals about why peer online mental health forums are needed*

- What advice would you give to someone starting to design a peer online mental health forum – explore in depth and why i.e. mechanisms?
- What kind of training do you think moderators need?- explore in depth and why ie mechanisms? and how?
- what kind of ongoing support do moderators need? explore in depth- why ie mechanisms? and how?
- What information do you think commissioners and health professionals need about forums to invest their time / money?
- Ask if anything they would like to add to help us understand the impacts of peer online mental health forums
